# HER-3: hub for escape mechanisms

**DOI:** 10.18632/aging.100850

**Published:** 2015-11-18

**Authors:** Emily Capone, Pramudita R Prasetyanti, Gianluca Sala

**Affiliations:** MediaPharma s.r.l., Laboratory of Molecular Oncology, Center of Excellence on AgingUniversity of Chieti-Pescara, Chieti, Italy

**Keywords:** HER-3, targeted therapy, drug resistance

Over the past decade, we have witnessed a significant increase in the clinical development of targeted therapeutics for the treatment of cancer. Smart drugs have been designed to boost the activity of traditional chemotherapy by selectively blocking signaling pathways on which cancer cells rely. A common feature of all these drugs is that they, directly or indirectly, prevent AKT or ERK activation, i.e. those signaling kinases mainly involved in the control of the critical oncogenic programs, including proliferation, migration, invasion and survival. A large group of these compounds is now routinely used in the clinical setting, including EGFR and HER-2 inhibitors (Gefitinib, Erlotinib and Lapatinib), BRAF inhibitor (Vemurafenib) and MEK1/2 inhibitor (Trametinib) and monoclonal antibodies either naked or in the form of drug-conjugates, targeting HER-2 (Trastuzumab, Pertuzumab and T-DM1), EGFR (Cetuximab and Panitunumab) and VEGF (Bevacizumab). Along these approved compounds, many others are currently under development, including a large number of PI3K and BRAF/MEK inhibitors.

Despite promising results at both the preclinical and clinical level, researchers and clinicians very soon had to reduce their enthusiasms once they encountered the problem of acquired drug resistance. Indeed, it is now an accepted paradigm that the prolonged use of these kind of inhibitors, and the resulting block of corresponding signaling pathways often leads to the relief of physiological feedback control mechanisms. This results in the activation of compensatory signaling pathways to maintain the oncogenic signaling and thereby contribute to inhibitor resistance [1]. With this in mind, clinicians and scientists quickly convened that only combination therapies and personalized medicine strategies would be able to overcome drug resistance in cancer therapy. In this respect, studies from different groups have validated the concept that the HER-3 receptor is critically involved in drug resistance in multiple cancers, acting as the main hub for escape mechanisms. HER-3 receptor is the preferred dimerization partner for the HER-2 receptor, and initially considered a poor target for therapy due to its limited intrinsic kinase activity. However, over the past ten years the role of this receptor in tumor development and therapy resistance has become clear and is no longer matter of debate [2]. Recently, the pharmacological inhibition of both PI3K/AKT and BRAF/MEK axes has been shown to relieve the feedback suppression of HER-3 signaling and other receptor tyrosyne kinases (RTK)s, which attenuates the therapeutic response to these agents [3, 4]. Mechanistically, up-regulation of HER-3 pathway mainly occurs at transcriptional level and results in an increased expression of the receptor on the surface of the treated cells, as documented in melanoma, thyroid and breast cancer models. Thus, the upregulation of HER-3 expression and signaling serves as an adaptive pro-survival response to different inhibitors leading to drug resistance. In reciprocal manner, NRG-1, the most potent HER-3 ligand has been found to be released by tumor-associated stromal cells, further promoting the idea that the HER-3 activation is vital in sustaining tumor growth.

We have recently extended the evidence in support of a role for the HER-3 pathway in drug resistance by showing that this receptor is upregulated in response to vemurafenib in a model of patient-derived colon cancer stem-like cells (CSC)s with documented BRAFV600E mutation [5]. We found that exogenous NRG-1β is able to stimulate colon CSCs and rescue the anti-proliferative effects of vemurafenib through the activation of HER-3. Importantly, we demonstrated that ligand dependent HER-3 activation is increased in vemurafenib treated cells and that this can be blocked by EV20, a humanized HER-3 antibody developed by our group [6, 7]. This suggests that HER-3 inhibition may be required to prevent escape from vemurafenib treatment.

In summary, evidences accumulated by many research groups, including our own, indicate that concurrent inhibition of HER-3 and PI3K/MEK pathways can lead to an optimal inhibition of cancer growth. Several monoclonal antibodies targeting HER-3 are now being developed by major pharmaceutical companies and some of them entered clinical testing. Conclusive results expected from ongoing trials and from other future trials using combinations not yet investigated, will reveal the true potential of HER-3 inhibition in the clinical practice.
